# Accuracy of next‐generation wireless skin temperature sensors during exercise–heat stress

**DOI:** 10.1113/EP093856

**Published:** 2026-06-09

**Authors:** Aaron J. E. Bach, Norman R. Morris, Surendran Sabapathy, David N. Borg, Isabella Ennever, Pramod Sharma, Fergus K. O'Connor

**Affiliations:** ^1^ School of Health Sciences and Social Work Griffith University Gold Coast Queensland Australia; ^2^ Australia Centre for Precision Health and Technology Griffith University Gold Coast Queensland Australia; ^3^ Griffith Institute for Human and Environmental Resilience Griffith University Nathan Queensland Australia; ^4^ Allied Health Research Collaborative The Prince Charles Hospital Metro North Hospital and Health Service Chermside Queensland Australia; ^5^ School of Health University of the Sunshine Coast Sippy Downs Queensland Australia; ^6^ School of Pharmacy and Medicine Griffith University Gold Coast Queensland Australia; ^7^ School of Health, Psychological and Medical Sciences University of Southern Queensland Toowoomba Queensland Australia

**Keywords:** ambulatory monitoring, body temperature regulation, ergometry, hot temperature, reproducibility of results, wearable electronic device

## Abstract

Skin temperature is fundamental in characterising human thermoregulatory responses. Wired probes, although accurate, restrict movement and are impractical outside laboratory settings. The iButton (DS1922L; Maxim Integrated, USA) is a widely used wireless alternative but does not meet the precision recommended by ISO 9886 and permits only retrospective data retrieval. A recently developed wireless sensor, the eTemp Performance (BodyCAP, France), claims to address these limitations but has not been validated independently. Here, we compared the eTemp and iButton against a wired thermocouple reference (SST‐1; Physitemp, USA) during rest, exercise and recovery in the heat [mean (SD): 35.0 (0.5)°C, 40.6 (1.5)% relative humidity]. Twenty‐six adults (10 women) completed seated rest (15 min), cycling at fixed heat production (30 min) and passive recovery (15 min). Mean skin temperature was calculated using the Ramanathan four‐site formula. Across all periods, the eTemp showed a small negative bias (mean difference, −0.04°C; 95% limits of agreement, −0.33°C to 0.25°C; concordance correlation coefficient, 0.986), whereas the iButton showed a small positive bias (+0.11°C; 95% limits of agreement, −0.23°C to 0.46°C; concordance correlation coefficient, 0.974). The eTemp met all a priori acceptability thresholds for mean skin temperature. The iButton met the acceptability threshold for mean bias in all periods and was statistically equivalent to the reference; however, the 95% confidence interval on the upper limit of agreement marginally exceeded 0.5°C during exercise, recovery and overall. These findings provide the first independent validation of the eTemp Performance sensor and support the use of both wireless devices for mean skin temperature measurement during exercise–heat stress, notwithstanding the known limitations in accuracy of the iButton against the ISO 9886 standard.

## Abstract (highlights)

- **What is the central question of this study?**
  Is the newly developed eTemp Performance wireless skin temperature sensor a valid and equivalent alternative to the established iButton and wired thermocouple reference?
- **What is the main finding and its importance?**
  Both the eTemp Performance and iButton wireless sensors demonstrated acceptable mean bias and statistical equivalence with a wired thermocouple reference across all exercise conditions. The eTemp met all a priori acceptability criteria for mean skin temperature; the limits of agreement for the iButton marginally exceeded our conservative thresholds. This is the first independent validation of the eTemp Performance sensor, supporting the use of these wireless conductive sensors for monitoring skin temperature during exercise–heat stress.

## 1 INTRODUCTION

As the outermost boundary of the body, the skin is the site at which heat exchange occurs with the surrounding environment and, thus, plays a crucial role in human thermoregulation. Accordingly, skin temperature data are used in the assessment of health, disease, injury (Cramer et al., 2022) and thermal comfort (Frank et al., 1999) and in the estimation of mean body temperature when combined with core temperature (Colin et al., 1971). Beyond controlled laboratory settings, wireless temperature sensors have been adopted in field‐based and occupational settings (Ioannou et al., 2021), veterinary sciences (Lee et al., 2015), free‐living sleep science (Hasselberg et al., 2013) and for ambient environmental logging (Runkle et al., 2019), where cabled instrumentation is impractical.

Despite the widespread use of skin temperature across these disciplines, there is currently no recognised gold‐standard measurement criterion (MacRae, Annaheim et al., 2018), with thermistors or thermocouples most commonly serving as the reference device in validation studies (Bach, Stewart, Disher et al., 2015; James et al., 2014; Smith et al., 2010; van Marken Lichtenbelt et al., 2006). Conductive sensors applied directly to the skin remain the most common approach, offering a practical balance of accuracy and portability, although any device affixed to the skin surface will inevitably influence local heat exchange to some degree (James et al., 2014). Within this category, wired thermocouples offer high sensitivity and resolution within the physiological range and can achieve the ±0.1°C precision specified by international standards for the evaluation of thermal strain (International Organisation for Standardisation, 2016). However, wired systems tether participants to recording equipment, introducing issues such as cable management, sensor dislodgement during movement, and impracticality in field settings (Bach, Stewart, Disher et al., 2015; Smith et al., 2010).

Wireless conductive sensors overcome many of the aforementioned constraints. The Thermochron iButton (DS1921H; Maxim Integrated, USA) has become the predominant wireless option for measurement of skin temperature in exercise and thermoregulatory research (Bandiera et al., 2026). Agreement between wired probes and iButtons has been examined across a range of conditions (Bach, Stewart, Disher et al., 2015; James et al., 2014; Smith et al., 2010; van Marken Lichtenbelt et al., 2006), with reported biases that tend to be within acceptable limits at rest but can widen during periods of rapid thermal (e.g., heat exposure) and physical (e.g., sweat, adhesion) change during exercise and recovery. The manufacturer‐stated accuracy of the iButton is ±0.5°C, which falls outside the ±0.1°C ‘precision’ recommended by ISO 9886 (International Organisation for Standardisation, 2016). Moreover, the iButton platform is no longer being developed actively and does not support real‐time data transmission, limiting its application for live monitoring.

The eTemp Performance (BodyCAP, France) is a newly developed, commercially available wireless skin temperature sensor with a manufacturer‐stated accuracy of ±0.1°C. A non‐commercial prototype BodyCAP skin sensor has been compared against the iButton during cycling in the heat (Bandiera et al., 2026); however, the recently marketed eTemp Performance device is yet to be assessed independently. Given that new measurement devices require independent validation before they can be used with confidence in research or practice, such an evaluation is warranted. The purpose of this study was to compare the accuracy and equivalence of the eTemp Performance and iButton wireless skin temperature sensors with a wired thermocouple reference, at individual anatomical sites and for mean skin temperature during rest, exercise and recovery in the heat in a controlled laboratory environment.

## 2 MATERIALS AND METHODS

### 2.1 Ethical approval

This study was approved by the Griffith University Human Research Ethics Committee (2025/805). The study conformed to the standard set by the *Declaration of Helsinki*, except for registration in a database. Verbal and written informed consent were obtained from all participants prior to commencing any experimental procedures.

### 2.2 Participants

A convenience sample of 27 healthy, physically active, non‐smoking adults were recruited; one individual withdrew following the familiarisation session, leaving 26 participants (16 men and 10 women; Table 1) who completed the study. Of the 10 women, two were using a combined oral contraceptive pill, two had a non‐hormonal copper intrauterine device, four were not using any form of contraception (one with regular natural cycles, one with oligomenorrhoea, and two with secondary amenorrhoea), and one had recently discontinued a hormonal intrauterine device (<1 month prior). Hormonal contraceptive use and menstrual status are reported in line with current methodological recommendations for sport and exercise science research involving women (Elliott‐Sale et al., 2021; Hutchins et al., 2021; Mee & Flood, 2026).

**TABLE 1:** Participant characteristics.

Participants	Age (years)	Height (cm)	Mass (kg)	BSA (m^2^)	*P*_max_ (W)	HR_max_ (beats min^−1^)	${\dot{V}}_{O_{2}\mathrm{peak}}$ (L min^−1^)	${\dot{V}}_{O_{2}\mathrm{peak}}$ (mL kg^−1^ min^−1^)	*P*_trial_ (W)
Men (*n* = 16)	26(6)	179(5)	84(17)	2.02(0.19)	331(50)	190(11)	3.92(0.82)	47.4(8.5)	148(34)
Women (*n* = 10)	26(4)	167(3)	63(6)	1.71(0.08)	252(42)	188(11)	2.73(0.41)	43.2(5.8)	118(11)
All (*n* = 26)	26(5)	174(8)	76(17)	1.90(0.22)	301(61)	189(11)	3.46(0.90)	45.8(7.7)	137(31)

*Note*: Data are reported as the mean (SD).

Abbreviations: BSA, body surface area; HR_max_, maximum heart rate; *P*_max_, peak power output; *P*_trial_, mean power during exercise in the heat trial; ${\dot{V}}_{O_{2}\mathrm{peak}}$, peak oxygen uptake.

Eligibility criteria required participants to be aged 18–35 years, pass Stage 1 and 2 of the Exercise and Sport Science Australia Adult Pre‐Exercise Screening System, have no thermoregulatory disorders, not be taking medications affecting thermoregulation or cardiovascular function, not be pregnant or planning pregnancy, have no adhesive allergies or skin conditions at sensor placement sites, and be capable of completing maximal exercise testing on a cycle ergometer.

An a priori sample size calculation was conducted to determine the number of participants needed to test whether the limits of agreement (LoA) fell within the maximal allowable interval of 0°C ± 0.5°C, accounting for the study design (i.e., a minimum of 59 repeat measurements on an individual within a period, i.e., rest, exercise or recovery). We specified a mean difference (bias) of 0.2 (Bach, Stewart, Disher et al., 2015), an SD (of the difference) of 0.1 (Smith et al., 2010), 95% LoA, and a 95% confidence level. Based on these parameters, a sample size of 22 gave us 80.4% power to conclude that a device is in agreement with the reference. We oversampled by 20% (*n* = 5) in case of participant attrition or missing data. The sample size calculation was made using the R package *SimplyAgree* (Caldwell, 2022).

### 2.3 Study design

This was an observational, validation study comparing two conductive wireless skin temperature sensor systems (eTemp and iButton) against a more traditional conductive wired reference thermocouple (Physitemp). Three sensor types were affixed simultaneously at four anatomical sites during rest, exercise and recovery in the heat. Each participant attended the laboratory on two occasions: (1) a preliminary session for familiarisation and maximal exercise testing; and (2) the main experimental trial.

### 2.4 Skin temperature devices

**FIGURE A1:**
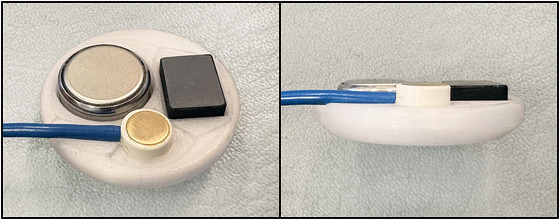
Device orientation and three‐dimensional printed housing for consistent placement and pressure between sensors.

Wired skin surface thermocouples (6.4 mm diameter × 4 mm height, cable 1.5 m; SST‐1 Skin Surface Probe; Physitemp Instruments, Clifton, NJ, USA) served as the criterion reference device. These sensors were connected to a Grant Squirrel 2020 data logger (Grant Instruments, UK). The manufacturer‐stated accuracy is ±0.1°C, with a resolution of 0.01°C across a range of 0°C–90°C (Figure A1).

The eTemp Performance (BodyCAP, France) are small (12.4 mm × 9.5 mm × 5.8 mm, ∼1.3 g), rechargeable, waterproof wireless skin temperature sensors that log continuously and can transmit data to a receiving device in real time via Bluetooth Low Energy. The manufacturer‐stated accuracy is ±0.1°C, with a resolution of 0.01°C across a range of −30°C to 50°C (Figure A1).

Thermochron iButtons (DS1922L; Maxim Integrated, USA) are small (17.4 mm diameter × 5.9 mm height), self‐contained, temperature data loggers that store data internally for subsequent download. The iButtons were programmed at a resolution of 0.0625°C. The manufacturer‐stated accuracy is ±0.5°C across the operating range of −40°C to +85°C (Figure A1).

**FIGURE A2:**
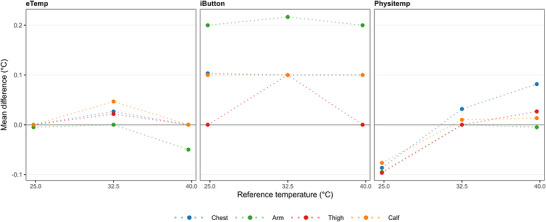
Mean error (measured minus reference) for each individual sensor across the three temperatures (25°C, 32.5°C and 40°C; *n* = 4 per device type). Each point is the mean of a 60 s sampling window in a stirred water bath.

Prior to data collection, all three types of sensors were verified against a NIST‐certifiable Type T thermocouple (Lutron TM‐947SD; precision, ±0.1°C) in a circulated water bath at three temperatures (25°C, 33°C and 40°C) spanning a normal physiological range. All devices returned values within their respective manufacturer‐stated accuracy specifications (Figure A2).

### 2.5 Sensor placement

All three sensor types were affixed simultaneously at four anatomical sites: chest (right pectoralis major), upper arm (lateral right upper arm at mid‐deltoid), anterior thigh (right quadriceps at mid‐thigh) and lateral calf (right lateral calf at widest circumference). A custom three‐dimensionally printed housing was designed to hold all three sensor types in a standardised arrangement at each site (Figure A1). The housing positioned each device as close together as possible while maintaining uniform pressure and ensuring that the contact surface of each sensor was flat against the skin, despite their differing dimensions. Skin sites were cleaned with alcohol swabs prior to sensor attachment. The housing and sensors were then attached to the skin using two strips of overlapping medical adhesive tapes (Elastoplast; Beiersdorf, Germany; Coban, 3M, USA).

### 2.6 Mean skin temperature calculation

Mean skin temperature (*T̅*_sk_) was calculated from the Ramanathan (1964) four‐site, weighted formula, given its acceptable agreement with higher‐site formulas during exercise in warm conditions (Bartman et al., 2025), as follows:

$${\bar{T}}_{sk}=0.3\times T_{chest}+0.3\times T_{arm}+0.2\times T_{thigh}+0.2\times T_{calf}$$

This formula was applied independently to each device type (Physitemp, eTemp and iButton) to generate device‐specific estimates of mean skin temperature at each time point.

### 2.7 Preliminary session

During the preliminary session (∼60 min), participants were familiarised with all equipment and experimental procedures. Participants then undertook a submaximal cycling bout on an electronically braked cycle ergometer (Excalibur Sport; Lode BV, Groningen, The Netherlands), where workload was adjusted until 5 min of stable metabolic data (measured continuously using indirect calorimetry; Ergocard CPX, MGC Diagnostics, USA) were obtained at the target heat production of between 6 and 7 W kg^−1^, in accordance with the approach of fixed heat production described by Cramer & Jay (2014). The workload identified during this session served as the starting intensity for the main experimental trial. After 10–15 min of rest, a maximal incremental exercise test was performed, beginning at a moderate intensity and increasing by 10–25 W every minute until voluntary exhaustion.

### 2.8 Experimental protocol

Participants were instructed to avoid alcohol and caffeine for 12 h, avoid strenuous exercise for 24 h, maintain normal hydration, and eat a light meal 2–3 h prior to the main experimental trial.

The main experimental trial began in an air‐conditioned room maintained at ∼22°C, where sensors were attached and a general‐purpose disposable rectal probe (12FR; DeRoyal Industries, USA) connected to a data logger (T‐TEC7; Temperature Technology, Australia) recording at 1 s intervals was self‐inserted to 15 cm by participants to monitor core body temperature. Heart rate and rhythm were monitored continuously (five‐lead ECG; Cardioline, Italy) during exercise. The group mean (SD) workload was 137 (31) W, and the mean steady‐state relative heat production during exercise (minutes 6–30 of the exercise period) was 6.3 (1.1) W kg^−1^.

The protocol consisted of three sequential periods. Initially, participants entered a climate chamber [mean (SD): 35.0 (0.5)°C and 40.6 (1.5) % relative humidity] and sat passively for 15 min. Following the rest period, participants mounted the electronically braked cycle ergometer, where they completed 30 min of continuous cycling exercise, with gas exchange measured as described above to maintain the target heat production. Following exercise cessation, participants resumed seated rest in the same climate chamber for 15 min of passive recovery.

All skin temperature devices recorded data continuously, at 2 s intervals. Core temperature was recorded at 1 s intervals and environmental conditions at 15 s intervals (HM70; Vaisala, Finland). All data were subsequently averaged into 1 min epochs for analysis. A brief transfer period occurred between the rest and exercise periods (mounting the bike and affixing indirect calorimetry), with a mean (SD) duration of 180 (102) s (range, 60–420 s). Data obtained during the transfer period were excluded from all analyses.

### 2.9 Statistical analysis

All analyses were performed in R (R Core Team, 2022) using the RStudio environment (v.2023.06.0; RStudio Team, 2020). All visualisations were generated using *ggplot2* (Wickham, 2016). The data and R code can be accessed from github.com/ajebach/tsk_validation. Missing data were inspected visually to check for systematic patterns of missingness (Borg et al., 2022). Differences were calculated as test device minus reference (i.e., eTemp minus Physitemp).

A priori acceptability thresholds were a mean difference between devices within 0.2°C and 95% LoA within 0.5°C (Bach, Stewart, Minett et al., 2015). Agreement was assessed using Bland–Altman analysis, with repeated‐measures 95% LoA to account for multiple observations on each participant (Bland & Altman, 2007). The 95% confidence intervals for the bias and LoA were computed using the MOVER method (Zou, 2013) via *SimplyAgree* (Caldwell, 2022). Lin's concordance correlation coefficient [CCC; and associated 95% confidence interval (CI)] was calculated as a single‐index measure of agreement (Lin, 1989). Finally, robust linear mixed‐effects models (Koller, 2016) were used to determine whether each device was statistically equivalent to the reference, by modelling the difference between each device and the reference. Period, time and period × time were included as fixed effects, and a random intercept term was included for each participant in the study to account for the correlation between repeated observations on an individual. Statistical equivalence was determined based on the 90% CI (Lakens et al., 2018) of the fitted values falling within the bounds of zero (Bach, Stewart, Minett et al., 2015) across all time points, within a period.

## 3 RESULTS

### 3.1 Missing data

**FIGURE A3:**
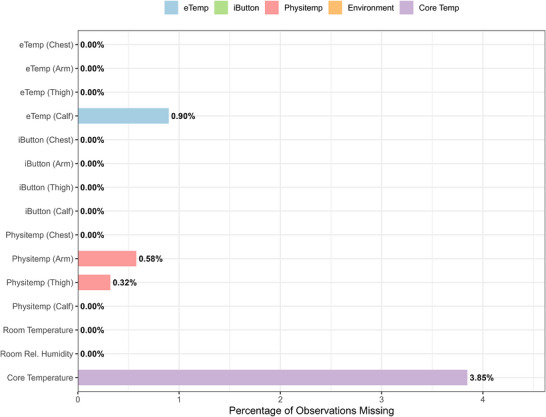
Missing data (as percentages) by variable.

**FIGURE A4:**
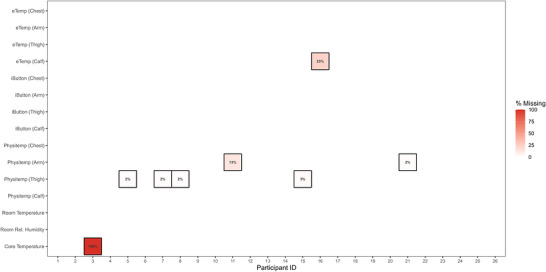
Missing data (as percentages) by variable and participant.

Missingness was extremely low (Figures A3 and A4). The iButton had complete data at all sites. The eTemp had 14 missing observations at the calf (0.9%), attributable to sensor detachment in one participant. The Physitemp had nine missing observations at the arm (0.6%) and five at the thigh (0.3%). One participant declined the rectal temperature probe; therefore, core temperature and mean body temperature are reported for *n* = 25. Where individual site data were missing, mean skin temperature was not calculated for that time point.

### 3.2 Mean skin temperature

The eTemp device demonstrated acceptable agreement with the Physitemp reference for mean skin temperature at rest, during exercise and recovery, and across all periods combined (Table 2). For all periods, the eTemp showed a small negative bias that was well within the a priori set threshold of 0.2°C, and the 95% CI on the LoA were within our threshold of 0.5°C. Concordance was excellent (≥0.947), and the eTemp device was statistically equivalent to the Physitemp reference in all periods.

**TABLE 2:** Comprehensive agreement analysis for the eTemp and iButton vs. Physitemp reference

Site	Period	MD (°C)	MD 95% CI	95% LoA (°C)	LoA 95% CI	CCC	CCC 95% CI	Equiv.
**eTemp**								
Mean *T*_sk_	**Overall**	**−0.04**	**[−0.07, −0.02]**	**[−0.33, 0.25]**	**[−0.36, 0.27]**	**0.99**	**[0.98, 0.99]**	**Yes**
	Rest	−0.03	[−0.07, 0.01]	[−0.29, 0.23]	[−0.35, 0.29]	0.98	[0.97, 0.98]	Yes
	Exercise	−0.00	[−0.04, 0.03]	[−0.28, 0.27]	[−0.32, 0.31]	0.97	[0.97, 0.98]	Yes
	Recovery	−0.14	[−0.18, −0.10]	[−0.41, 0.14]	[−0.47, 0.19]	0.95	[0.94, 0.96]	Yes
Chest	**Overall**	**−0.04**	**[−0.09, 0.01]**	**[−0.43, 0.34]**	**[−0.49, 0.41]**	**0.97**	**[0.97, 0.97]**	**Yes**
	Rest	−0.07	[−0.13, 0.00]	[−0.44, 0.31]	[−0.55, 0.41]	0.95	[0.94, 0.96]	Yes
	Exercise	−0.00	[−0.06, 0.05]	[−0.37, 0.37]	[−0.45, 0.44]	0.94	[0.93, 0.95]	Yes
	Recovery	−0.10	[−0.16, −0.03]	[−0.50, 0.30]	[−0.59, 0.40]	0.93	[0.92, 0.94]	Yes
Arm	**Overall**	**−0.05**	**[−0.09, 0.00]**	**[−0.48, 0.38]**	**[−0.53, 0.44]**	**0.97**	**[0.97, 0.97]**	**Yes**
	Rest	−0.09	[−0.16, −0.03]	[−0.45, 0.27]	[−0.55, 0.36]	0.97	[0.97, 0.98]	Yes
	Exercise	0.03	[−0.03, 0.09]	[−0.37, 0.44]	[−0.46, 0.52]	0.94	[0.93, 0.95]	Yes
	Recovery	−0.15	[−0.23, −0.07]	[−0.59, 0.29]	[−0.72, 0.42]	0.92	[0.91, 0.94]	Yes
Thigh	**Overall**	**−0.04**	**[−0.10, 0.01]**	**[−0.53, 0.44]**	**[−0.59, 0.51]**	**0.98**	**[0.97, 0.98]**	**Yes**
	Rest	0.03	[−0.08, 0.13]	[−0.51, 0.56]	[−0.68, 0.73]	0.94	[0.93, 0.95]	Yes
	Exercise	−0.03	[−0.08, 0.03]	[−0.44, 0.39]	[−0.52, 0.46]	0.97	[0.96, 0.97]	Yes
	Recovery	−0.15	[−0.24, −0.06]	[−0.66, 0.36]	[−0.80, 0.50]	0.91	[0.89, 0.93]	Yes
Calf	**Overall**	**−0.05**	**[−0.10, 0.00]**	**[−0.49, 0.39]**	**[−0.55, 0.45]**	**0.98**	**[0.98, 0.98]**	**Yes**
	Rest	0.05	[−0.01, 0.11]	[−0.29, 0.39]	[−0.38, 0.48]	0.97	[0.96, 0.97]	Yes
	Exercise	−0.04	[−0.09, 0.02]	[−0.43, 0.35]	[−0.51, 0.43]	0.97	[0.96, 0.97]	Yes
	Recovery	−0.18	[−0.27, −0.09]	[−0.68, 0.32]	[−0.82, 0.47]	0.93	[0.91, 0.94]	Yes
**iButton**								
Mean T_sk_	**Overall**	**+0.11**	**[0.07, 0.16]**	**[−0.23, 0.46]**	**[−0.28, 0.51]**	**0.97**	**[0.97, 0.98]**	**Yes**
	Rest	+0.11	[0.07, 0.16]	[−0.16, 0.39]	[−0.23, 0.45]	0.97	[0.96, 0.97]	Yes
	Exercise	+0.14	[0.09, 0.19]	[−0.21, 0.48]	[−0.27, 0.55]	0.93	[0.92, 0.94]	Yes
	Recovery	+0.06	[−0.01, 0.13]	[−0.32, 0.45]	[−0.43, 0.56]	0.94	[0.93, 0.95]	Yes
Chest	**Overall**	**+0.07**	**[−0.00, 0.14]**	**[−0.41, 0.54]**	**[−0.51, 0.64]**	**0.95**	**[0.95, 0.96]**	**Yes**
	Rest	+0.06	[−0.02, 0.13]	[−0.37, 0.48]	[−0.49, 0.60]	0.95	[0.93, 0.96]	Yes
	Exercise	+0.08	[0.01, 0.16]	[−0.38, 0.55]	[−0.49, 0.66]	0.90	[0.88, 0.91]	Yes
	Recovery	+0.04	[−0.06, 0.14]	[−0.52, 0.60]	[−0.68, 0.76]	0.89	[0.87, 0.91]	Yes
Arm	**Overall**	**+0.20**	**[0.13, 0.26]**	**[−0.32, 0.71]**	**[−0.40, 0.79]**	**0.94**	**[0.93, 0.95]**	**Yes**
	Rest	+0.10	[0.03, 0.16]	[−0.27, 0.47]	[−0.38, 0.57]	0.97	[0.97, 0.98]	Yes
	Exercise	+0.28	[0.21, 0.35]	[−0.18, 0.73]	[−0.27, 0.83]	0.84	[0.82, 0.86]	Yes
	Recovery	+0.13	[0.01, 0.25]	[−0.51, 0.76]	[−0.71, 0.96]	0.88	[0.86, 0.90]	Yes
Thigh	**Overall**	**+0.08**	**[0.02, 0.14]**	**[−0.43, 0.59]**	**[−0.51, 0.67]**	**0.97**	**[0.97, 0.98]**	**Yes**
	Rest	+0.13	[0.04, 0.22]	[−0.34, 0.60]	[−0.47, 0.73]	0.95	[0.93, 0.96]	Yes
	Exercise	+0.11	[0.03, 0.18]	[−0.38, 0.60]	[−0.49, 0.71]	0.94	[0.94, 0.95]	Yes
	Recovery	−0.03	[−0.13, 0.07]	[−0.58, 0.52]	[−0.73, 0.67]	0.92	[0.90, 0.93]	Yes
Calf	**Overall**	**+0.10**	**[0.02, 0.18]**	**[−0.45, 0.65]**	**[−0.57, 0.76]**	**0.97**	**[0.96, 0.97]**	**Yes**
	Rest	+0.20	[0.13, 0.27]	[−0.18, 0.58]	[−0.28, 0.68]	0.93	[0.91, 0.94]	Yes
	Exercise	+0.05	[−0.05, 0.14]	[−0.52, 0.61]	[−0.66, 0.75]	0.93	[0.92, 0.94]	Yes
	Recovery	+0.09	[−0.02, 0.21]	[−0.52, 0.71]	[−0.70, 0.89]	0.91	[0.89, 0.93]	Yes

*Notes*: Tsk calculated using the four‐siteweighted formula of Ramanathan (1964); limits of agreement calculated using themethods of Bland & Altman (2007) and Caldwell (2022); LoA 95% CI calculatedusing the method of Zou (2013); CCC calculated using the method of Lin (1989).

Abbreviations: 95% LoA (°C), 95% limits of agreement; CCC, Lin's concordance correlation coefficient with 95% CIs; Equiv., equivalence; LoA 95% CI, most upperand most lower bound of overall limits of agreement; MD 95% CI, confidence intervals of themean difference; MD, mean difference (test minus reference); Tsk, mean skin temperature.

Compared with the reference, iButtons showed an acceptable mean bias (small positive) in all periods, falling within our acceptability threshold of 0.2°C, excellent concordance (≥0.931), and were statistically equivalent (Table 2). However, the agreement did not meet our acceptability threshold for LoA overall or during exercise or recovery, because the 95% CI upper bound on the LoA for these periods extended marginally beyond ±0.5°C (overall, 0.51°C; exercise, 0.55°C; and recovery, 0.56°C; Table 2). At rest, the iButton mean bias and LoA fell within our acceptability thresholds (Table 2).

### 3.3 Individual site agreement

**FIGURE 1:**
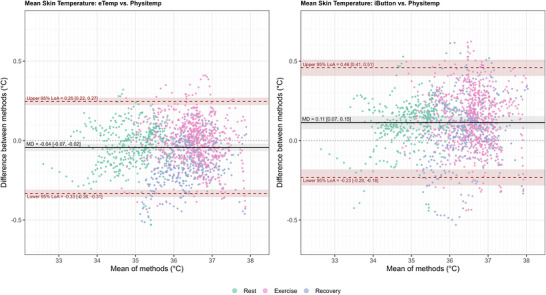
Bland–Altman plots (Bland & Altman, 2007) of mean skin temperature (in degrees Celsius) differences for the eTemp (top) and iButton (bottom) compared with the Physitemp reference thermocouple. Individual data points represent minute‐averaged observations, coloured by experimental period (rest, exercise and recovery). The continuous black line indicates the mean difference (bias), and the dashed red lines indicate the repeated‐measures 95% limits of agreement (Caldwell, 2022). Shaded grey ribbons represent the 95% confidence interval for the bias, and shaded red ribbons represent the 95% confidence intervals for the upper and lower limits of agreement (Zou, 2013).

**FIGURE A5:**
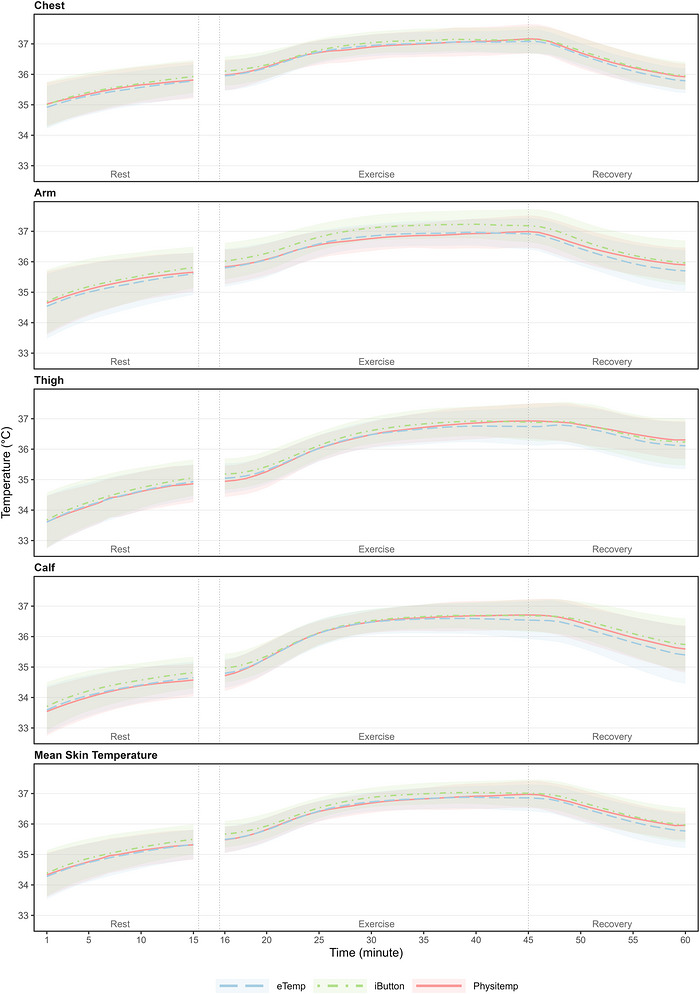
Time series of at each site and mean skin temperature responses (in degrees Celsius) (lines = mean, ribbons = SD; *n* = 26).

**FIGURE A6:**
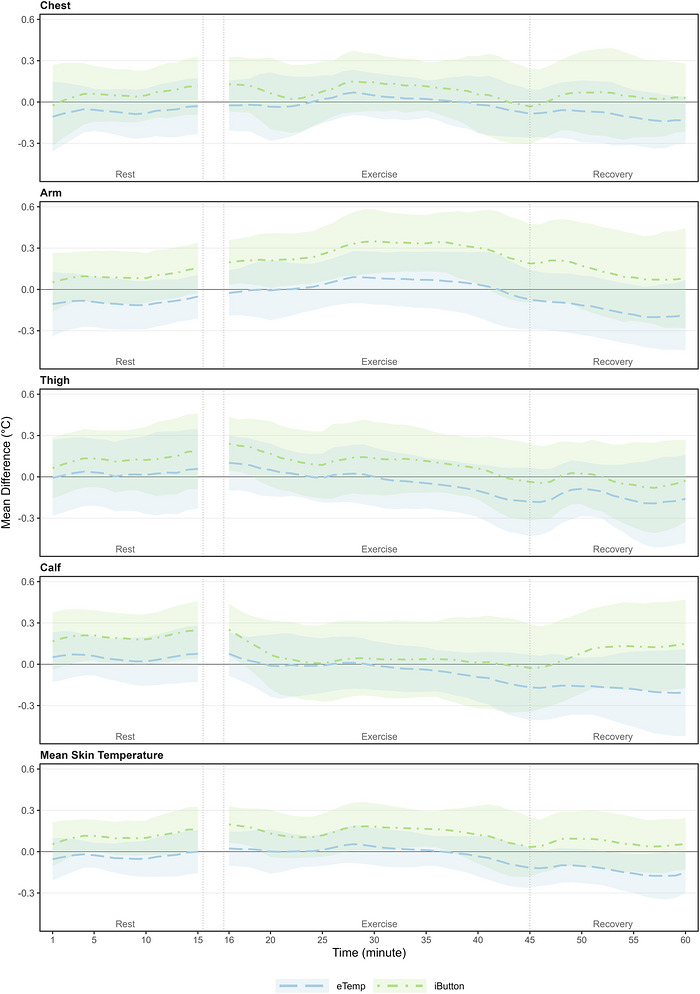
Time series of mean differences (in degrees Celsius) between eTemp or iButton and the Physitemp reference (lines = mean, ribbons = SD; *n* = 26).

**FIGURE A7:**
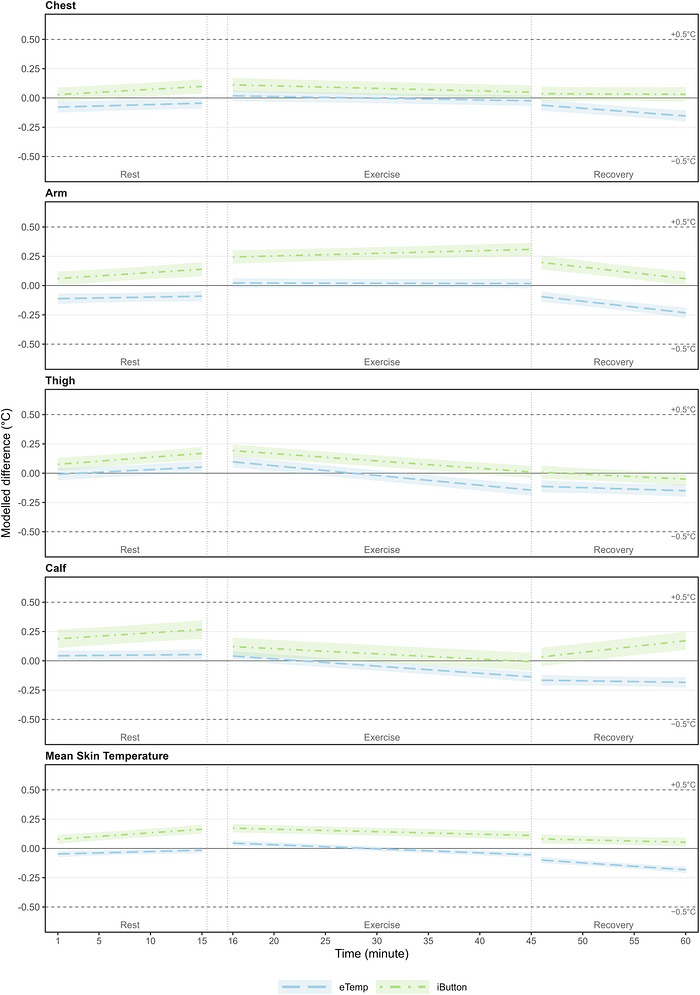
Robust linear mixed‐effects model fitted differences (in degrees Celsius) between eTemp or iButton and Physitemp reference over time (lines = fitted mean, ribbons = 90% confidence interval). Dashed horizontal lines indicate the ±0.5°C equivalence bounds; all modelled 90% confidence interval ribbons within bounds indicate statistical equivalence.

At the individual site level, the eTemp demonstrated consistent small negative biases across all four sites (mean difference range, −0.04°C to −0.05°C; Figure 1; Table 2). Mean bias was acceptable overall and at all sites during rest and exercise. Mean bias at the arm, thigh and calf exceeded 0.2°C during recovery. The 95% LoA exceeded 0.5°C at all sites during all periods, with the exception of the chest during exercise and the calf at rest. Concordance was excellent across all sites and peiods (CCC range, 0.92–0.98), and the eTemp was statistically equivalent to the reference at all sites across all periods (Figure A5, A6, A7).

The iButton demonstrated acceptable mean bias at the chest (overall and across all periods), the arm at rest, the thigh (overall, during exercise and during recovery) and the calf (overall and during exercise). The 95% LoA exceeded ±0.5°C at all sites and periods. Concordance was generally excellent, although it was lowest at the arm during exercise (CCC = 0.84) and recovery (CCC = 0.88). The iButton was statistically equivalent to the reference at all sites across all periods (Figure A7).

## 4 DISCUSSION

In this study, we compared the eTemp Performance and iButton wireless skin temperature sensors against a wired thermocouple reference during rest, exercise and recovery in the heat. For mean skin temperature, both devices met the a priori set acceptability criteria for mean bias, with mean differences within 0°C ± 0.2°C during all periods. Only the eTemp device had LoA that fell within our acceptability criteria of 0°C ± 0.5°C, across all periods. The iButton LoA fell, by a very small margin (range, 0.02°C–0.06°C), outside our acceptability threshold overall or during periods of dynamic changes in mean skin temperature (exercise and recovery). These findings provide the first independent validation of the eTemp Performance sensor, supporting its use for measurement of mean skin temperature during exertional heat stress. Although the iButton did not meet our conversative criteria (LoA within 0.5°C), it falls well within the less conservative limits reported by similar studies for assessing mean skin temperature in the heat (Bach, Stewart, Disher et al., 2015; James et al., 2014; MacRae, Rossi et al., 2018; Smith et al., 2010; van Marken Lichtenbelt et al., 2006).

The eTemp demonstrated closer overall agreement with the reference than the iButton at the mean skin temperature level, with a near‐zero bias and narrower limits of agreement. At individual sites, both devices showed small, directionally consistent biases, with the eTemp tending towards slight underestimation and the iButton towards slight overestimation, although the magnitude of bias was more variable across sites for the iButton. Smith et al. (2010) reported iButton–thermistor differences of 0.26°C–0.32°C during passive rest and exercise at 30°C. Bach, Stewart, Disher et al. (2015) found iButton biases against a wired reference ranging from near zero at rest (+0.01°C) to +0.26°C during exercise in the heat and +0.37°C during recovery. Importantly, although the two devices showed biases in opposite directions relative to the reference, mean bias for both devices was well within the a priori threshold of 0.2°C across all periods, and the observed differences are unlikely to be of practical significance for most applications.

Although the eTemp demonstrated acceptable agreement for mean skin temperature, several individual anatomical site–period combinations had limits of agreement that extended beyond the acceptability interval of −0.5°C to 0.5°C. This result can be explained simply by reduced variance in temperature when averaging across the four anatomical sites, using the weighted equation described by Ramanathan (1964). The variance at individual sites is explained, at least in part, by localised differences in skin blood flow, regional morphology and sweat distribution (Maniar et al., 2015; Taylor & Notley, 2018). Where local skin temperature at a single site is of interest, practitioners should consider the suitability of these devices depending on their intended application.

**FIGURE A8:**
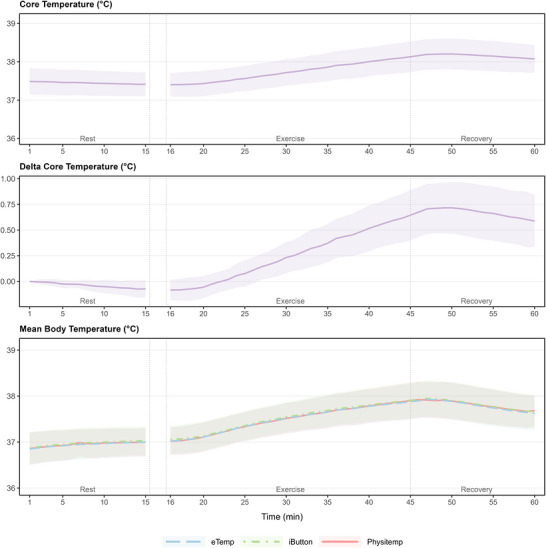
Time series of core and mean body temperature responses (in degrees Celsius) (lines = mean, ribbons = SD; *n* = 25). Mean body temperature was calculated as follows: 0.8 × core temperature + 0.2 × mean skin temperature.

Variance can be reduced further when mean skin temperature is combined with core temperature to estimate mean body temperature, where the weighting applied to mean skin temperature can range from 0.10 to 0.36 depending on the formula and conditions used (Colin et al., 1971; Jay et al., 2010). Under a weighting of 0.2 (Colin et al., 1971), a mean skin temperature variance of 0.1°C would contribute only ∼0.02°C to the estimated mean body temperature. At the level of agreement demonstrated by both devices in this study, the downstream impact on derived variables, such as mean body temperature, is negligible for most research purposes (Figure A8).

Beyond measurement accuracy, practical considerations might also inform device selection. The iButton stores data internally for *post hoc* retrieval and has a long deployment history. Many of the disadvantages of the iButton (e.g., non‐rechargeable, retrospective data retrieval) have been overcome with the eTemp Performance, which can function as both a data logger and a real‐time transmitter. Data are stored in the internal memory of the device regardless of whether a receiving device is in range, hence temporary signal loss does not result in missing data; stored data are downloaded the next time the sensor comes in range of its receiver. Together, these features extend the practical reach of skin temperature measurement beyond the laboratory and more readily into real‐time monitoring in field‐based contexts, such as occupational, athletic and remote or prolonged deployments where wired instrumentation is impractical. The agreement reported here, however, reflects a single, controlled laboratory trial during short‐duration exercise–heat stress. Field deployment introduces additional considerations not assessed in the present study, including clothing, mechanical disturbance and diverse environmental conditions outside the validated range (e.g., solar radiation, cold, high air velocity).

**FIGURE A9:**
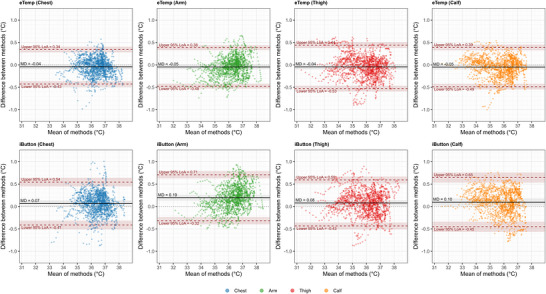
Bland–Altman plots temperature differences (in degrees Celsius) at individual sites for the eTemp (top) or iButton (bottom) compared with the Physitemp reference thermocouple. Individual data points represent minute‐averaged observations for all participants (*n* = 26). The continuous black line indicates the mean difference (bias), and the dashed red lines indicate the repeated‐measures 95% limits of agreement. Shaded grey ribbons represent the 95% confidence interval for the bias, and shaded red ribbons represent the 95% confidence intervals for the upper and lower limits of agreement.

**FIGURE A10:**
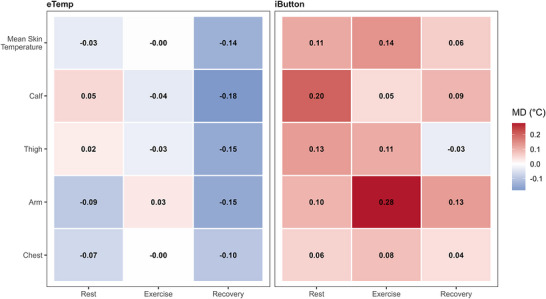
Heatmap of mean differences (MD) in temperature (in degrees Celsius) from reference by site and mean skin temperature.

The findings of this study should be interpreted in light of several limitations. The study was conducted in a single, hot–dry environmental condition (35°C and 40% relative humidity). Skin temperature becomes increasingly heterogeneous in cold environments as peripheral vasoconstriction creates larger temperature gradients across the body surface (Webb, 1992). The relatively homogeneous skin temperature distribution in the heat means that the four‐site formula used and the co‐location of three sensors at each site are well justified for the present conditions (Bartman et al., 2025). However, device performance and the suitability of this measurement approach might differ in cooler or more thermally asymmetric environments, where regional skin temperature variability is greater. The three‐dimensionally printed housing used to co‐locate all three sensor types ensured that agreement was assessed at effectively the same anatomical site under uniform contact pressure, but the combined sensor assembly is larger than any single device in typical use (Figure A1). The extent to which this influenced the local skin microenvironment and, consequently, absolute temperature readings is unknown. However, any such effect would apply equally to all devices and is unlikely to have biased the between‐device comparisons (Figure A9, A10).

## 5 CONCLUSION

Both the eTemp Performance and iButton wireless skin temperature sensors demonstrated acceptable mean bias and statistical equivalence with a wired thermocouple reference during rest, exercise and recovery in the heat. The eTemp met all a priori acceptability criteria for mean skin temperature, including LoA within ±0.5°C across all periods. The LoA for the iButton exceeded this threshold marginally during exercise and recovery. The eTemp showed closer agreement with the reference overall, although both devices performed well within the measurement precision thresholds needed for most thermoregulatory research and applied monitoring. On the basis of these findings, the eTemp Performance can be recommended as a valid wireless sensor for measurement of mean skin temperature during exercise–heat stress, and the iButton remains a suitable alternative where the observed accuracy limitations are acceptable.

## AUTHOR CONTRIBUTIONS

Study conception and design: Aaron J. E. Bach, Fergus K. O'Connor, Norman R. Morris, Surendran Sabapathy and Pramod Sharma. Data collection: Aaron J. E. Bach, Fergus K. O'Connor. and Isabella Ennever. Analysis and interpretation of results: Aaron J. E. Bach. and David N. Borg. Drafting the manuscript: Aaron J. E. Bach, David N. Borg. and Fergus K. O'Connor. All authors have read and approved the final version of this manuscript and agree to be accountable for all aspects of the work in ensuring that questions related to the accuracy or integrity of any part of the work are appropriately investigated and resolved. All persons designated as authors qualify for authorship, and all those who qualify for authorship are listed.

## CONFLICT OF INTEREST

Aaron Bach and Fergus O'Connor are members of the Editorial Board for *Temperature*. Fergus O'Connor is an Editor for *Applied Physiology, Nutrition, and Metabolism* and *Discover Public Health*. Norman Morris is on the Editorial Board for *Respiratory Physiology & Neurobiology*. Surendran Sabapathy is an Associate Editor for *Frontiers in Physiology*. David Borg is an Associate Editor for *Communications in Kinesiology*, *Journal of Sports Sciences* and *Research Quarterly for Exercise and Sport*. All authors have no other conflicts of interest to declare.

## FUNDING INFORMATION

No funding was received for this study.

## Supporting information



Supplementary Information

## Data Availability

The anonymised data are provided as Supporting Information to this paper. Additionally, the anonymised data and R code used for analysis can be accessed from github.com/ajebach/tsk_validation.
